# Emotion anticipation and processing in depression: Behavioral, neural, and physiological reactivity

**DOI:** 10.1192/j.eurpsy.2025.10043

**Published:** 2025-06-11

**Authors:** Magdalena Wlad, Wiebke Struckmann, Jonas Persson, Jörgen Rosén, David Fällmar, Robert Bodén, Malin Gingnell

**Affiliations:** 1 Department of Medical Sciences, Psychiatry, Uppsala University, Uppsala, Sweden; 2 Department of Psychiatry and Behavioral Sciences, Stanford University, Stanford, CA, USA; 3 Department of Clinical Neuroscience, Karolinska Institute, Solna, Sweden; 4 Department of Surgical Sciences, Neuroradiology, Uppsala University, Uppsala, Sweden; 5 Department of Psychology, Uppsala University, Uppsala, Sweden

**Keywords:** anterior cingulate cortex, depression, electrodermal activity, functional magnetic resonance imaging, insula, salience network

## Abstract

**Background:**

Depression is characterized by disturbed emotion processing, with aberrant neural and physiological responses to emotional stimuli. Here, we applied an emotion anticipation and processing paradigm to investigate brain neural and electrodermal reactivities in patients with depression compared with healthy controls.

**Methods:**

The study included 42 patients (27 females) and 44 healthy controls (21 females). Subjects underwent functional magnetic resonance imaging with simultaneous measurement of electrodermal activity. During scanning, red or green color cues were presented, followed by pictures of negative or positive valence, respectively. Behavioral valence and arousal ratings of the picture stimuli were conducted after scanning. Anhedonia was assessed through a semi-structured interview in both subject groups.

**Results:**

Patients perceived positive pictures as less positive than controls did. Positive anticipation (i.e., green color cues) elicited stronger activations in the anterior cingulate cortex and the right insula in patients than in healthy controls, indicating salience network disturbances. An exploratory analysis of all regions in the Automated Anatomical Labeling Atlas 2 found significant differences in activity to positive anticipation between groups in several brain regions involved in cognition and emotion processing. Positive and negative anticipation elicited stronger electrodermal responses in healthy controls. However, electrodermal reactivity to negative pictures was higher in patients than in controls.

**Conclusions:**

Ongoing depression affects emotion anticipation and processing at the behavioral, neural, and physiological levels. These findings contribute to increased understanding of the disorder.

## Introduction

Depression, a common and debilitating psychiatric disorder, is characterized by disturbed emotion processing [1]. Core symptoms include feelings of worthlessness or hopelessness, negative interpretations of internal and external stimuli, and an inability to experience positive emotions. This negativity bias has also been shown in studies measuring valence ratings of emotional stimuli, with more negative valence ratings of sad faces and neutral images in unipolar and bipolar depression, respectively [2, 3]. Anhedonia, that is, reduced ability to experience pleasure, is a common symptom in depression and can include decreased consummatory pleasure (reflecting the momentary pleasure experienced during an enjoyable activity) and decreased anticipatory pleasure (reflecting pleasure experienced from anticipating future activities).

The underlying biological mechanism of depression, especially in individual cases, is still largely unknown, despite extensive research on the subject. Functional magnetic resonance imaging (fMRI) studies of unipolar depression have found aberrant neural activation in cortico-limbic circuits in response to negative emotional stimuli, such as hyperactivity in the amygdala and insula [4–7]. Hyperactivity in the insula to positive stimuli has been reported in depression [8, 9]. Studies have also reported increased activation in the subgenual anterior cingulate cortex (ACC) in response to positive stimuli and decreased activation in the pregenual ACC and dorsal ACC in response to negative stimuli in unipolar/bipolar depression [10]. Activation of the ACC has also been associated with catastrophizing and worrying [11]. It seems that mere anticipation of emotional stimuli also evokes different neural responses in depression, such as hyperactivity in the amygdala [12] and attenuated responses in the prefrontal cortical regions [13], although studies investigating emotion anticipation, as opposed to emotion processing, are fewer in comparison. Studies investigating reward anticipation and processing have found aberrant responses in depression [14, 15].

The depressive state is associated not only with changes in neural circuits but also with dysregulation of the sympathetic and parasympathetic nervous systems. Well over a 100 years ago, psychologists suggested that bodily signals play an important part in the experience of emotions. The James–Lange theory [16] proposed that peripheral, bodily changes precede the emotional experience, and that emotions are experienced when these peripheral changes are interpreted by the brain. More recent neuroimaging research has supported this theory and suggested that the insula, in particular, substantializes subjective feelings from bodily signals and provides emotional awareness [17], possibly through a posterior-to-mid-to-anterior integration of interoceptive information in the insula [17, 18]. There is also evidence that physiological signals feedback to the brain and influence cognition and decision-making [19]. One physiological signal of importance is electrodermal activity (EDA; also referred to as skin conductance). A systematic review [20] found that many studies report hypoactive electrodermal baseline activity and lower electrodermal responses to emotional stimuli in depressive states, although elevated electrodermal reactivity to emotional stimuli [21] and during fear conditioning [22] has also been reported.

In summary, the abovementioned studies have found that depression is characterized not only by changes in emotion processing at the behavioral level but also by aberrant neural reactivity to emotional stimuli and by changes in sympathetic activity. However, how these changes relate to and affect each other is largely unknown, and more research is warranted. Finding and understanding biomarkers of depressive disorders could improve our diagnostics and treatment of them. In this study, we aimed to link previous findings by investigating emotion anticipation and processing at the behavioral, neural, and physiological levels in patients with depression compared with healthy controls.

Considering the clinically described negativity bias and anhedonia in depression, we expected patients to perceive negative stimuli as being more negative and positive stimuli as being less positive than controls would. We expected to find differences in neural reactivity to both positive and negative anticipation, and positive and negative stimuli in patients compared with controls. Based on previous findings of hyperactivity in the amygdala and insula to negative stimuli in depression, we specifically hypothesized that patients would show higher neural reactivity to negative anticipation and negative stimuli in limbic regions compared with healthy controls. In line with reports of electrodermal hypoactivity in depression, we expected to find a lower electrodermal response to our emotional task in patients.

## Materials and Methods

### Participants

This cross-sectional study contains baseline data from a randomized clinical trial investigating intermittent theta burst stimulation (iTBS) treatment for depression [23] and an add-on brain-imaging study [24] with shared methodology conducted at the Brain Stimulation Unit, Uppsala University Hospital, Sweden. Patients were recruited from the psychiatric outpatient clinic at the Uppsala University Hospital. All patients met criteria for an ongoing unipolar or bipolar depressive episode as verified through a Mini International Neuropsychiatric Interview (M.I.N.I.) [25] and scored ≤40 points on the Motivation and Pleasure Scale-Self-Report [26]. Patients between 18 and 59 years of age were eligible for inclusion. Patients’ medication had to be unchanged 1 month before the study start. Exclusion criteria comprised pregnancy, epilepsy, metal implants, and active substance use disorder. Sample size was determined based on the power analyses conducted in the original treatment study [23].

In addition, healthy controls aged 18–59 were recruited through online advertisements. In addition to the previously mentioned exclusion criteria, these healthy controls were required not to have any ongoing or history of psychiatric disorder as verified through an M.I.N.I. interview.

Written informed consent was obtained from all participants upon entering the study. The study was approved by the Ethical Review Board, Uppsala, Sweden.

In total, 42 patients and 44 healthy controls were included in the study.

### Study design

Treatment with iTBS was performed after the collection of imaging data and started on the following weekday after baseline assessments (clinical ratings and fMRI scanning), as described in Reference [24]. Participants underwent fMRI scanning with simultaneous recordings of EDA. Morphological images from all subjects were reviewed by a senior consultant in neuroradiology to exclude malformations and significant parenchymal defects. Symptoms of anhedonia were assessed using the Clinical Interview for Assessment of Negative Symptoms (CAINS) [27], which discriminates between consummatory and anticipatory pleasures [28, 29]. Recent research has shown that the CAINS can be used to assess negative symptoms in depression [30]. Consummatory and anticipatory pleasure rates were calculated as the sum of CAINS items 3 and 8, and 4, 6, and 9, respectively. Each item was rated on a scale from 0 to 4; increasing scores indicate increasing severity of anhedonia.

### Emotion anticipation and processing paradigm

The paradigm used in this study consisted of a task targeting both emotion anticipation and emotion processing, previously described by Gingnell et al. [31]. Stimuli were 15 pictures of positive valence and 15 pictures of negative valence, selected from the International Affective Pictures System (IAPS) [32] and presented to the participants in a pseudorandomized order on a display to induce emotion processing. The pictures were matched on valence and arousal. Each picture was preceded by a color cue indicating the valence of the subsequent picture to induce emotion anticipation. The color cues were red slides for negatively valenced pictures and green slides for positively valenced pictures. Participants were informed of this before scanning. The color cues were presented for 5 s, followed by a black screen with a jittered interval of 2.5–3.5 s, after which a picture was shown for 2 s, and finally a black screen was presented for 11 s. Participants were instructed to look at the screen; no motor or verbal response throughout the experimental session was required. Participants viewed all 30 pictures again outside the scanner room immediately after the scanning session and rated valence and arousal for each picture on the Self-Assessment Manikin used in the IAPS material. Valence was rated on a scale from 1 to 9, ranging from highly negative to highly positive. Arousal was similarly rated on a scale from 1 to 9, ranging from low arousal to high arousal.

### fMRI and EDA data acquisition

All 42 patients and 44 healthy controls underwent fMRI scanning; EDA data were recorded for 41 patients and 44 healthy controls. fMRI scanning took place at Uppsala University Hospital, Sweden. Imaging was performed using a 3T scanner (Philips Achieva, Best, The Netherlands) with a 32-channel head coil. Details regarding the scanner and parameter settings are provided in Supplementary Material S1.

EDA was recorded during the fMRI task using electrodes attached to the palmar surface of the hypothenar eminence of the left hand. Details regarding materials and data acquisition settings are provided in Supplementary Material S1. For most participants, high-frequency artifacts were filtered out using a hardware high-pass filter during recording, while for a few participants, no high-pass filter was used due to setting changes in the recording system.

### Data analysis

#### fMRI data analysis

Preprocessing of the fMRI data was performed through fMRI prep [33, 34]. The description of the preprocessing of both anatomical and functional data, including references, is provided in Supplementary Material S2.

#### EDA data analysis

Data were analyzed in Python (Python Software Foundation, USA) using PyCharm and the NeuroKit2 toolbox [35]. Due to data collection error where some subjects’ EDA signals were not recorded, only participants with signals more than 0.01 μS in more than half of their trials were included. Furthermore, participants were excluded from further analyses due to signal artifacts if more than half of their event-related Skin conductance responses (SCRs) had a value above 3 μS.

Data were normalized (the square root of each event-related SCR was computed) and mean range-corrected (the event-related SCR was divided by the subject’s mean value) [36] before a baseline correction analysis was conducted [37], where the highest skin conductance value within a time window after stimulus onset was identified. Baseline was calculated as the mean value between 0 and 0.5 s after stimulus onset. The mean value of the SCR response to the four contrasts, *green, red, negative* picture, and *positive* picture, was then computed for each participant and used in subsequent statistical analyses.

### Statistical analyses

Demographic data were compared between subject groups (patients and healthy controls) by two-tailed independent samples *t*-tests or *χ* ^2^-tests using MATLAB R2018b, with significance set at *p* < 0.05.

Valence and arousal ratings were compared between groups using two-sample *t*-tests in MATLAB R2018b. The significance threshold was set at *p* < 0.05.

Scores of consummatory and anticipatory pleasures, respectively, were compared in MATLAB R2018b between groups using two-sample *t*-tests and a significance threshold at *p* < 0.05.

Imaging data were analyzed as a region of interest (ROI) analysis. ROIs were chosen based on previous literature highlighting these regions as important in depression and consisted of the ACC, amygdala (right and left), and insula (right and left) as defined by the Automatic Anatomical Labeling (AAL) 2 and Talairach Daemon database atlas in the WFU Pickatlas toolbox for SPM [38–42]. Further details of the first- and second-level analyses are provided in Supplementary Material S3.

As an additional exploratory analysis, all brain regions defined in the AAL2 atlas [42] were compared between groups to analyze differences in neural reactivity to *negative* and *positive* pictures, as well as *red* and *green* color cues. Statistical significance was set at *p* < 0.05 False discovery rate (FDR-corrected).

Differences in electrodermal response between patients and controls were explored using two-sample *t*-tests with a significance level set to *p* < 0.05.

## Results

### Participants

Forty-two patients and forty-four healthy controls were included in the study. Demographic and clinical data are shown in Table 1. Patients with depression did not differ significantly from healthy controls on sociodemographic variables.

**Table 1.** Baseline demographic and clinical characteristics of healthy controls and patients

	Controls (*n* = 44)	Patients (*n* = 42)	Test for difference
Years of age, mean (SD)	29.6 (11.3)	29.2 (9.4)	*t*(84) = 0.16 *p* = 0.088
Sex, female:male	27:17	21:21	*χ* ^2^ (1) = 1.13, *p* = 0.288
Years of education, *n*			*χ* ^2^ (2) = 5.02, *p* = 0.081
Nine year completed	2	7	
Twelve year completed	31	21	
Higher education	11	14	
Primary diagnosis, *n*			
Depressive episode	–	25	
Recurrent depression	–	14	
Bipolar disorder	–	3	
Medication, *n*			
Antidepressants	–	36	
Mood stabilizers	–	12	
Antipsychotics	–	8	
Antihistamines^a^	–	8	
No medication	–	4	

a Except for one patient, the antihistamines were no standing prescriptions but only taken when needed.

SD, standard deviation.

### Behavioral ratings

Valence and arousal ratings showed that negative and positive pictures were experienced with the intended emotional valence in both patients and controls, and that negative pictures induced higher arousal than positive pictures (see Table 2 and Supplementary Material S4). There was a statistically significant difference in valence ratings between patients and healthy controls, where patients perceived *positive* pictures as being less positive than controls did (*t*(84) = −4.25, *p* < 0.001) (see Table 2).

**Table 2.** Valence and arousal ratings (mean [standard deviation]) for negative and positive stimuli in the healthy control and patient group

	Controls (*n* = 44)	Patients (*n* = 42)	Test for difference
Negative valence	3.0 (0.7)	3.5 (1.2)	*t*(84) = 1.96, *p* = 0.054
Positive valence	7.0 (0.4)	6.0 (1.2)	*t*(84) = −4.25, *p* < 0.001
Negative arousal	5.6, (0.7)	5.2 (1.7)	*t*(84) = 1.41, *p* = 0.162
Positive arousal	4.5 (0.6)	4.0 (1.7)	*t*(84) = 1.36, *p* = 0.178

*Note*: Valence and arousal were each rated on a scale from 1 to 9, with 1 indicating negative valence or low arousal, respectively, and 9 indicating positive valence or high arousal, respectively.

CAINS ratings of anhedonia are presented in Table 3. CAINS scores for consummatory pleasure were missing from one healthy control. Confirming group designation, there were significant differences between groups regarding scores of consummatory and anticipatory pleasures, with higher anhedonia ratings in the patient group than in the control group.

**Table 3.** Scores of anhedonia symptoms, rated through subitems in the Clinical Assessment Interview for Negative Symptoms (CAINS)

	Controls (*n* = 43)	Patients (*n* = 42)	Test for difference
Consummatory pleasure, mean (SD)	0.80 (1.1)	4.8 (2.0)	*t*(83) = −11.8, *p* < 0.001

	Controls (*n* = 44)	Patients (*n* = 42)	Test for difference
Anticipatory pleasure, mean (SD)	0.8 (1.5)	7.5 (3.1)	*t*(84) = −12.8, *p* < 0.001

*Note*: Consummatory pleasure rates were calculated as the sum of Items 3 and 8. Anticipatory pleasure rates were calculated as the sum of Items 4, 6, and 9. Each item was rated on a scale from 0 (indicating no deficits) to 4 (indicating severe deficits); increasing scores indicate increasing severity of anhedonia.

SD, standard deviation.

### fMRI data

**Figure 1.**
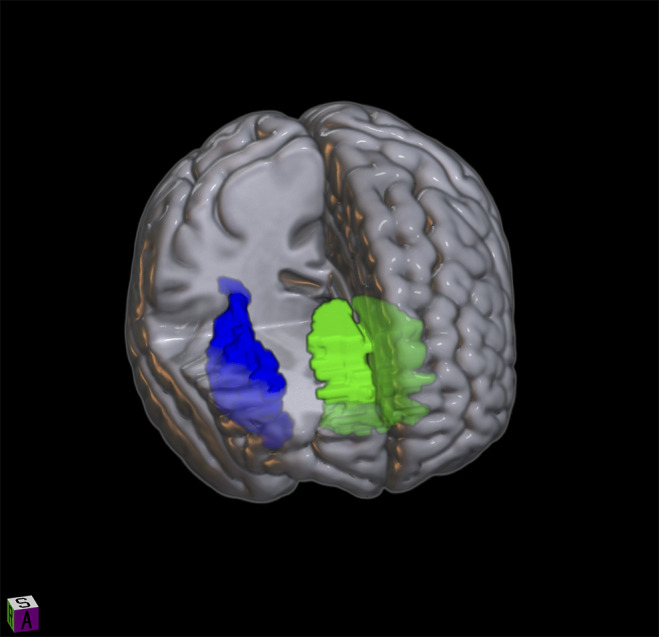
The bilateral ACC (green) and right insula (blue), where patients had increased activation to positive anticipation compared with controls. Image created using MRIcroGL from http://www.nitrc.org/.

**Figure 2.**
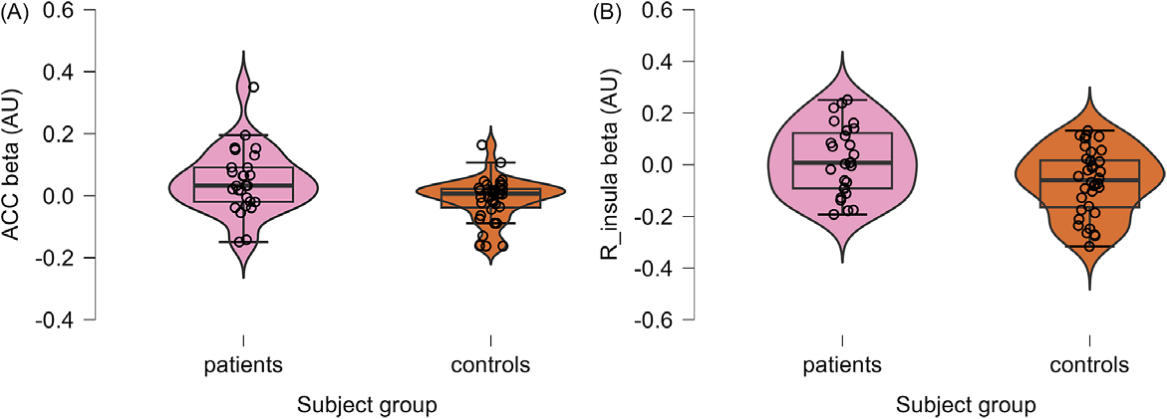
(A) Violin chart depicting neural activity in the anterior cingulate cortex (ACC) to positive anticipation. The plot shows extracted beta weights (arbitrary units [AUs]) for patients (pink) and controls (orange). The boxplot shows the minimum value (excluding outliers), the first quartile, the median value, the third quartile, and the maximum value (excluding outliers). Individual data points below the boxplot’s minimum value or above the boxplot’s maximum value are outliers. The width of the violin plot indicates the density of data points. (B) Violin chart depicting neural activity in the right insula to positive anticipation. The plot shows extracted beta weights (arbitrary units [AUs]) for patients (pink) and controls (orange). The boxplot shows the minimum value (excluding outliers), the first quartile, the median value, the third quartile, and the maximum value (excluding outliers). Individual data points below the boxplot’s minimum value or above the boxplot’s maximum value are outliers. The width of the violin plot indicates the density of data points.

fMRI data were excluded due to excessive motion artifacts for three patients and three healthy controls. In addition, data were excluded for 12 patients and 5 healthy controls due to missing data. This resulted in fMRI data from 27 patients and 36 healthy controls. Comparisons between patients and healthy controls in the ROI analyses revealed a higher activation in the bilateral ACC (*t*(62) = −2.48, *p*_FDR_ = 0.043) in patients (mean = 0.046, SD = 0.1) than in controls (mean = −0.012, SD = 0.07) in the *green* contrast. Furthermore, results showed higher right insula activation (*t*(62) = −2.53, *p*_FDR_ = 0.043) in patients (mean = 0.017, SD = 0.13) than in controls (mean = −0.067, SD = 0.13) in the *green* contrast (see Figures 1 and 2A,B and Tables 4, 5).

**Table 4.** Activation of the anterior cingulate cortex (ACC) to positive anticipation

	ACC *β* (AU)	
	Patients	Controls
Median	0.032	0.006
Mean	0.046	−0.012
95% CI mean upper	0.087	0.012
95% CI mean lower	0.005	−0.036
Standard deviation	0.104	0.070
IQR	0.110	0.060
Minimum	−0.149	−0.163
Maximum	0.350	0.164

**Table 5.** Activation of the right insula to positive anticipation

	Right insula *β* (AU)	
	Patients	Controls
Median	0.007	−0.060
Mean	0.017	−0.067
95% CI mean upper	0.069	0.024
95% CI mean lower	−0.036	−0.110
Standard deviation	0.133	0.126
IQR	0.214	0.182
Minimum	−0.192	−0.316
Maximum	0.251	0.132

*Note:* Extracted β-values are shown.

AU, arbitrary units.

Results from the exploratory analysis of all regions in the AAL2 atlas are shown in Supplementary Material S5. In summary, 20 brain regions from the AAL2 atlas were identified where neural activity differed between patients and healthy controls. Differences in neural activity were found only for the *green* contrast.

### EDA data

EDA data were recorded for 41 patients and 44 healthy controls. For 19 patients and 31 healthy controls, electrodermal responses did not exceed 0.01 μS in more than half of their trials, and these were not included in the main analysis but were later included in an additional analysis (see Supplementary Material S6). The main analysis, therefore, included 22 patients and 13 healthy controls. Data were missing for one healthy control in the supplementary analysis in one *green* and one *positive* picture trial; the remaining data from this subject were included in the analysis. For 20 patients and 12 healthy controls, high-frequency artifacts were filtered out using a hardware high-pass filter during recording, while for 2 patients and 1 healthy control, no high-pass filter was used.

Patients differed significantly from healthy controls in their electrodermal reactivity. Healthy controls had a higher electrodermal reactivity to *red* and *green* color cues (*t*(33) = 2.70, *p* = 0.011 and *t*(33) = 2.67, *p* = 0.012, respectively), while patients had a significantly higher reactivity to *negative* pictures (*t*(33) = −2.54, *p* = 0.016). No statistically significant difference between groups was found for reactivity to *positive* pictures.

## Discussion

In this study, we explored emotion anticipation and processing at the behavioral, neural, and physiological levels in depression to link previous findings of disturbed emotional processes in this disorder. We indeed found that patients with depression differ from healthy controls at all three levels.

### Behavioral changes in emotion processing in depression

We found that both patients and controls perceived negative pictures as being more negative than positive pictures, confirming the intended valence of the paradigm stimuli. As hypothesized, patients perceived positive pictures as less positive than controls did, which is in line with previous studies [43], as well as the reported experienced deficits in feelings of anticipatory and consummatory pleasure, the core features of anhedonia.

### Aberrant neural responses to emotion anticipation and processing in ongoing depression

Confirming our hypothesis, we found several differences in neural activity between patients with an ongoing depressive episode and healthy controls. Positive anticipation (i.e., green color cues) elicited higher activation in the bilateral ACC and the right insula in patients.

The ACC has a key role in emotion processing through its regulatory role with respect to limbic regions and also seems to have an important role in the appraisal and expression of negative emotion [44]. Activation of the ACC has been associated with the appraisal of anxiety-provoking potential threats [45], as well as with catastrophizing and worrying [11]. Aberrant ACC activity in depressive states has been described in previous studies [10, 46, 47]. Elevated ACC activity in response to positive stimuli has been reported in depression [10], although in our study we found an increased activity to positive *anticipation* and not positive stimuli per se. These findings indicate abnormal functioning of this brain region and are in line with the key role of the ACC in emotion processes that seem to be disturbed in an ongoing depression. An abnormally increased activity could indicate maladaptive or less effective signaling between important brain regions involved in emotion regulation.

The insula is involved in the detection and processing of emotionally salient stimuli [48, 49] as well as interoceptive awareness [50] and integration of visceral and sensory information [51]. The right anterior insula mediates interoceptive awareness and activity in this brain region is also correlated with interoceptive accuracy [50]. Aberrant insular activity has previously been reported in depression with respect to interoception [52]. In a review article, Sliz and Hayley argued that disrupted interoception and an inability to exert cognitive control over emotionally relevant information in depressive states could be reflected in aberrant insular activity [51]. Another study found lower amygdala–insula connectivity during an emotion regulation task in adolescents with unipolar depression, suggesting that poor emotion regulation in depression may be due to poorer insular integration of signals from the amygdala [53]. Our findings of increased insular activity support the theory of disrupted emotional processing and interoception in depression. Previous studies have found an increase in insular activity to positive stimuli in unipolar depression [8, 9]; however, in our study, aberrant insular activity was found for positive anticipation only.

Both the ACC and insula are part of the salience network (SN) [54], which is involved in the integration and detection of internal and external salient information. Aberrant activity in the SN in unipolar depression has been described previously [5, 55]. The right insula, in particular, has been suggested to have a key role in contributing to appropriate behavioral responses to salient stimuli by switching between central-executive and default-mode networks [56]. Our finding of aberrant right insular and ACC activity in response to positive anticipation further adds evidence to SN disturbances in depression. Moreover, the ACC and insula are often co-activated during emotional experiences [17], and although the insula instantiates subjective feelings from bodily signals and feelings of emotion, it has been proposed that the ACC integrates interoceptive information and provides the volitional agent that can modulate these feelings [17, 18, 50]. This model thereby challenges the criticism of the James–Lange theory that argued that this theory did not allow for feelings of internally generated emotion [17]. Contrary to our hypothesis, we did not find any differences in neural activity in response to negative anticipation between groups. Abler et al. found increased activation in the amygdala during anticipation of aversive stimuli in unipolar depression [12], albeit using symbols of emotional content as emotional cues, which could perhaps affect the results.

Also contrary to our hypothesis, we did not find any differences in neural reactivity during emotional *processing* (i.e., positive and negative stimuli) between patients and controls. This was somewhat surprising, especially considering that several other studies report aberrant neural reactivity to emotional stimuli in depression, and that we indeed see a lower EDA response to emotional stimuli in the present study. Elevated amygdala reactivity to negative stimuli has been reported in some [4, 57] but not all [58–60] depression studies; one study found increased amygdala reactivity only in bipolar, but not unipolar, depression [61]. While these somewhat inconsistent findings might reflect methodological differences, such as types of stimuli, illness severity, and medication status, one also has to consider that neural responses to cued stimuli can differ from those to noncued stimuli, as anticipation itself might moderate the response, as has been shown in previous studies in healthy subjects [62].

Our exploratory analysis showed differences in neural reactivity to positive anticipation between patients and controls in several brain regions. These included regions involved in cognitive processes, such as the precuneus [63], the caudate nucleus [64], the middle [65], superior [66], and inferior [67, 68] frontal gyri, as well as regions important for emotional processes, such as the cingulate gyrus [69]. We also found differences in parts of the cerebellum that have previously been proposed to monitor and synchronize the main networks involved in both cognitive and emotional processes [70], as well as in the temporal pole, which is associated with cognitive and socio-emotional processing [71]. In addition, we found differences in the olfactory cortex, part of the olfactory system [72], and the hippocampus, involved in memory consolidation [69]. Several of the abovementioned brain regions are also part of the limbic system, which has an important role in emotional, motivational, and memory processes [69]. Although we are hesitant to draw conclusions from these findings due to the exploratory nature of the analysis, we suggest that several brain regions implicated in cognitive and emotional processing are affected in depression. These regions could be of interest to study further in future neuroimaging studies.

### Aberrant physiological responses to emotion anticipation and processing in depression

In line with our hypothesis, we found aberrant electrodermal responses in depression compared with healthy controls. Controls had a significantly higher electrodermal reactivity to red and green color cues, that is, negative and positive anticipation, compared with patients. Previous studies have indeed found hypoactive electrodermal responses in depressive states [20], although results tend to vary depending on the emotional stimuli. For example, Tsai et al. [73] found that individuals with unipolar depression showed a lower electrodermal response in comparison with healthy controls during sad and amusing film clips. However, during fear conditioning, higher EDA responses to conditioned stimuli than to nonconditioned stimuli have also been reported for unipolar depression [22]. This might explain our second finding of higher reactivity in the patient group to negative pictures, which include both sad and fear-provoking items.

Some studies have suggested that electrodermal responses may vary in different subtypes of depression [20]. If this is the case, it would make our results more difficult to interpret since all patients were analyzed as one group.

These findings contribute to a deeper understanding of depressive disorders as not only affecting central but also peripheral, neural circuits and peripheral signaling in emotional processes.

Interestingly, aberrant responses to positive anticipation were found in the patient group at the behavioral (deficits in anticipatory pleasure), neural (increased activity in the ACC and insula), and physiological (lower electrodermal response) levels. As previously mentioned, the insula substantializes subjective feelings from bodily signals and provides emotional awareness, while the ACC integrates interoceptive information and modulates those feelings. An increase in insular and ACC activity despite lower sensory input from the peripheral sympathetic nervous system could be interpreted as evidence of disrupted sensory integration and interoception in depression. This could be one possible explanation for the disturbances in emotion processing seen in depression.

### Limitations

This study is subject to limitations. First, the sample size was only moderate for a brain imaging study, especially since several subjects had to be excluded due to motion artifacts and missing data. One way to minimize the issue of head movement could be to use increased levels of physical restraint; however, this could make the study less feasible for the participants. We included not only patients with unipolar depression but also three patients with bipolar depression in our study, which could affect some of our results. For example, Thorell et al. found that the prevalence of electrodermal hyporeactivity was higher in patients with bipolar (80%) as compared with unipolar depression (67%) [74]. Previous neuroimaging studies have found differences in neural activity between unipolar and bipolar depression, including lower ACC activation in unipolar [75] and elevated amygdala activity in bipolar depression [61]. However, studies directly comparing the two conditions are scarce, making it difficult to draw conclusions. We also included patients with psychiatric comorbidities (anxiety disorders and Attention Deficit Hyperactivity Disorder (ADHD)/Attention Deficit Disorder (ADD)), which could potentially affect our results, although subsamples with a specific comorbidity were too small to perform separate analyses, especially after exclusion of data due to motion artifacts and missing data. On the other hand, a transdiagnostic approach is representative of the clinical reality and could make our results more generalizable to the general clinical patient population.

Moreover, colorblindness was not an exclusion criterion in our study.

Another limitation is that our study design did not include a neutral stimulus condition; therefore, the different conditions are contrasted against an implicit baseline. Although this approach has been used in other studies [46, 76, 77], it would provide more information on the mechanisms behind aberrant neural reactivity in depression to also include a neutral condition. However, adding a neutral condition would lengthen the paradigm and could make the study less feasible for participants.

Several participants in the patient group were treated with antidepressants, mood stabilizers, antipsychotics, and antihistamines (see Table 1), which could influence our results on neural and electrodermal reactivity. As discussed in a review article by Wandschneider and Koepp, pharmaco-fMRI studies have indeed shown that common antidepressant medications such as selective serotonin reuptake inhibitors can lead to attenuation of the limbic/paralimbic regions and increased activation in prefrontal networks [78]. A recent study on lamotrigine found reduced neural reactivity in a network of regions associated with emotion processing, such as the amygdala, insula, and ACC, to an emotional task, albeit in healthy volunteers [79]. Other studies in healthy volunteers have found an effect of lithium on neural activity and connectivity in an emotion regulation task [80], but no effect of quetiapine on emotional processing other than during recognition memory [81]. When it comes to antidepressant medication and its effect on EDA, results are inconclusive; some studies find no correlations, whereas others report reduced EDA in medicated subjects [20], making it difficult to draw conclusions. Administration of quetiapine during acute anxiety in specific phobias decreases electrodermal reactivity during exposure [82].

It should also be noted that one of the inclusion criteria for the study was that the patients had not received a satisfactory effect from their pharmacological treatment. Therefore, it is possible that the presence of medication might not be as pronounced in creating a bias in our group as when comparing treatment responders to healthy individuals. However, our belief is that the relatively small sample size does not allow for reliable assessments of the effect of medication status within the present study.

Finally, many subjects had to be excluded from our main EDA analysis due to low signals/data signal collection errors. However, results were similar when these subjects were included in the supplementary analysis, except that the group difference in reactivity to negative pictures did not persist.

In conclusion, we studied emotion anticipation and processing in ongoing depression using different methods to link proposed disturbances at the behavioral, neural, and physiological levels. At the behavioral level, clinical assessments and valence ratings of picture stimuli indicated anhedonia in the patient group. We also found increased ACC and insula (both parts of the SN) reactivity to positive anticipation in depression, highlighting these regions as important in emotion regulation and adding insight into the underlying mechanism of depressive symptoms. Furthermore, we found aberrant physiological reactivity to both emotion processing and anticipation, suggesting that depression not only affects central neural circuits but also peripheral signaling. In addition, an increase in insular activity despite lower sensory input from the peripheral sympathetic nervous system might reflect disrupted interoception in depression. Our findings contribute to existing knowledge of the disturbances in emotion regulation that are characteristic of depression.

## Supporting information

10.1192/j.eurpsy.2025.10043.sm001 Wlad et al. supplementary material Wlad et al. supplementary material

## Data Availability

Due to sensitivity reasons, the data supporting the findings of this study are not publicly available, but could be available from the corresponding author upon reasonable request.

## Supplementary material

The supplementary material for this article can be found at http://doi.org/10.1192/j.eurpsy.2025.10043.
