# Supplementary material for: Emotion anticipation and processing in depression: Behavioral, neural, and physiological reactivity
Source: Eur Psychiatry. 2025 Jun 11;68(1):e68. doi: 10.1192/j.eurpsy.2025.10043 (PMC12188339; doi:10.1192/j.eurpsy.2025.10043)
Supplement: Wlad et al. supplementary material [file S0924933825100436sup001.docx]

**Supplementary Material**

**S1. fMRI and electrodermal activity data acquisition***fMRI data acquisition*Functional T2*-weighted images were obtained using echo planar imaging (EPI) (repetition time (TR)= 2s, echo time (TE)= 30ms, flip angle= 90, field of view (FOV)= 64x64 mm² with voxel size= 3x3x3 mm³, 300 dynamic scans acquired, interleaved acquisition with transverse slice orientation). T1-weighted structural imaging was performed using a 3D multi-shot spin echo sequence (TR= 8.2ms, TE=3.8ms, flip angle= 8, FOV= 256x256 mm² with voxel size= 1x1x1 mm³).

*Electrodermal activity data acquisition*The electrodes used for data acquisition were two Ag- AgCl electrodes (EL509 Biopac electrodes) filled with isotonic electrode gel (GEL101 Biopac gel). Skin conductance responses (SCR) were recorded with a sampling rate of 1000 Hz. Data was recorded using the Biopac MP150 system and Acqknowledge software, version 4.2 (BIOPAC systems, Goleta, CA, USA).

**S2. fMRI data analysis**

The description of the preprocessing of the imaging data was automatically generated by fMRI prep 23.2.1 [1, 2] and is included here verbatim, as advocated by the developers ([fMRIPrep: A Robust Preprocessing Pipeline for fMRI Data — fmriprep version documentation](https://fmriprep.org/en/stable/)), but with small stylistic changes. Results included in this manuscript come from preprocessing performed using fMRIPrep 23.2.1 [1, 2], which is based on Nipype 1.8.6 [3, 4].

*Anatomical data preprocessing*T1-weighted (T1w) images were found within the input BIDS dataset. The T1w image was corrected for intensity non-uniformity (INU) with N4BiasFieldCorrection [5], distributed with ANTs 2.5.0 [6], and used as T1w-reference throughout the workflow. The T1w-reference was then skull-stripped with a Nipype implementation of the antsBrainExtraction.sh workflow (from ANTs), using OASIS30ANTs as target template. Brain tissue segmentation of cerebrospinal fluid (CSF), white-matter (WM) and gray-matter (GM) was performed on the brain-extracted T1w using fast (FSL (version unknown) [7]). Volume-based spatial normalization to one standard space (MNI152NLin2009cAsym) was performed through nonlinear registration with antsRegistration (ANTs 2.5.0), using brain-extracted versions of both T1w reference and the T1w template. The following template was selected for spatial normalization and accessed with TemplateFlow (23.1.0, [8]): ICBM 152 Nonlinear Asymmetrical template version 2009c [(Fonov, 2009), TemplateFlow ID: MNI152NLin2009cAsym].

*Functional data preprocessing*

For each of the 1 BOLD runs found per subject (across all tasks and sessions), the following preprocessing was performed. First, a reference volume was generated, using a custom methodology of fMRIPrep, for use in head motion correction. Head-motion parameters with respect to the BOLD reference (transformation matrices, and six corresponding rotation and translation parameters) are estimated before any spatiotemporal filtering using mcflirt (FSL, [9]). The BOLD reference was then co-registered to the T1w reference using mri_coreg (FreeSurfer) followed by flirt (FSL, [10]) with the boundary-based registration [11] cost-function. Co-registration was configured with six degrees of freedom. Several confounding time-series were calculated based on the preprocessed BOLD: framewise displacement (FD), DVARS and three region-wise global signals. FD was computed using two formulations following Power (absolute sum of relative motions, [12], and Jenkinson (relative root mean square displacement between affines, [9]). FD and DVARS are calculated for each functional run, both using their implementations in Nipype (following the definitions by [12]). The three global signals are extracted within the CSF, the WM, and the whole-brain masks. Additionally, a set of physiological regressors were extracted to allow for component-based noise correction (CompCor, [13]). Principal components are estimated after high-pass filtering the preprocessed BOLD time-series (using a discrete cosine filter with 128s cut-off) for the two CompCor variants: temporal (tCompCor) and anatomical (aCompCor). tCompCor components are then calculated from the top 2% variable voxels within the brain mask. For aCompCor, three probabilistic masks (CSF, WM and combined CSF+WM) are generated in anatomical space. The implementation differs from that of Behzadi et al. in that instead of eroding the masks by 2 pixels on BOLD space, a mask of pixels that likely contain a volume fraction of GM is subtracted from the aCompCor masks. This mask is obtained by thresholding the corresponding partial volume map at 0.05, and it ensures components are not extracted from voxels containing a minimal fraction of GM. Finally, these masks are resampled into BOLD space and binarized by thresholding at 0.99 (as in the original implementation). Components are also calculated separately within the WM and CSF masks. For each CompCor decomposition, the k components with the largest singular values are retained, such that the retained components’ time series are sufficient to explain 50 percent of variance across the nuisance mask (CSF, WM, combined, or temporal). The remaining components are dropped from consideration. The head-motion estimates calculated in the correction step were also placed within the corresponding confounds file. The confound time series derived from head motion estimates and global signals were expanded with the inclusion of temporal derivatives and quadratic terms for each [14]. Frames that exceeded a threshold of 0.5 mm FD or 1.5 standardized DVARS were annotated as motion outliers. Additional nuisance timeseries are calculated by means of principal components analysis of the signal found within a thin band (crown) of voxels around the edge of the brain, as proposed by [15]. All resamplings can be performed with a single interpolation step by composing all the pertinent transformations (i.e. head-motion transform matrices, susceptibility distortion correction when available, and co-registrations to anatomical and output spaces). Gridded (volumetric) resamplings were performed using nitransforms, configured with cubic B-spline interpolation. Many internal operations of fMRIPrep use Nilearn 0.10.2 ([16], mostly within the functional processing workflow. For more details of the pipeline, see the section corresponding to workflows in fMRIPrep’s documentation.

**S3. Statistical analysis of fMRI data**Parameters included in the first-level analysis were onsets and durations of negatively and positively valenced pictures, red and green color cues, black screens between color cue and picture, as well as movement parameters obtained from preprocessing. Realignment parameters (direction x y z, rotation x y z), aCompCor (1-6) and framewise displacement (FD) were entered as 13 separate regressors to control for movement. The individual beta-estimates for each condition were subsequently entered into contrasts (contrasted against an implicit baseline) to test for the effects of exposure (*negative* and *positive*) and anticipation (*red* and *green*). The contrast images from each participant were then used in second-level analyses at group level.
In the group-level analysis of the imaging data, simple t-tests were used to test whether the contrast estimate for each ROI was different from zero. Mean contrast estimates for every ROI were extracted with the help of the spm_read_vols.m function implemented in SPM12. T-tests were then performed on all extracted ROI estimates in R (R Core Team (2013), [R: The R Project for Statistical Computing (r-project.org)](https://www.r-project.org/)). The uncorrected p-values were then adjusted for false discovery rate (FDR) according to the method described by Benjamini and Yekutieli [17]. The level of significance was set to p < 0.05 (FDR-corrected). Extraction of mean contrast estimates was performed using the AAL atlas [18].

**S4. Valence and arousal ratings**
Patients (n=42) rated *negative* and *positive* pictures significantly different in their valence (t(41)=-7.85, *p*<.001), with lower valence attributed to *negative* (mean=3.5, SD=1.2) than to *positive* (mean=6.0, SD=1.2) pictures. For arousal ratings, patients rated *negative* and *positive* pictures significantly different (t(41)=4.679, *p*<.001), with higher arousal attributed to *negative* (mean=5.2, SD=1.7) than to *positive* (mean=4.0, SD=1.7) pictures.

Healthy controls (n=44) also rated *negative* and *positive* pictures significantly different in their valence (t(86)=18.7, *p*<.001), with lower valence attributed to *negative* (mean=3.0 SD=0.7) than to *positive* (mean=7.0 SD=0.4) pictures. For arousal ratings, healthy controls rated *negative* and *positive* pictures significantly different (t(85)=-3.8, *p*<0.001), with higher arousal attributed to *negative* (mean=5.6, SD=0.7) than to *positive* (mean=4.5, SD=0.6) pictures.

**S5. Exploratory ROI- analysis**

| **Nr.** | **Label** | **Anatomical brain region** | **t- statistic** |
| --- | --- | --- | --- |
| 40 | Frontal_Inf_Tri_R | Right inferior frontal gyrus, triangular part | 3.31 |
| 30 | Cingulate_Mid_R | Right middle cingulate and paracingulate gyrus | 3.31 |
| 47 | Frontal_Sup_Medial_L | Left superior frontal gyrus, medial | 3.24 |
| 46 | Frontal_Sup_2_R | Right superior frontal gyrus | 3.16 |
| 73 | Olfactory_L | Left olfactory cortex | 3.15 |
| 7 | Caudate_L | Left caudate nucleus | 3.14 |
| 38 | Frontal_Inf_Orb_2_R | Right inferior frontal gyrus, pars orbitalis | 3.11 |
| 45 | Frontal_Sup_2_L | Left superior frontal gyrus, dorsolateral | 3.09 |
| 108 | Temporal_Pole_Sup_R | Right temporal pole, superior temporal gyrus | 3.01 |
| 29 | Cingulate_Mid_L | Left middle cingulate and paracingulate gyrus | 2.99 |
| 90 | Precuneus_R | Right precuneus | 2.97 |
| 48 | Frontal_Sup_Medial_R | Right medial superior frontal gyrus | 2.93 |
| 37 | Frontal_Inf_Orb_2_L | Left inferior frontal gyrus, pars orbitalis | 2.91 |
| 44 | Frontal_Mid_2_R | Right middle frontal gyrus | 2.88 |
| 26 | Cerebelum_Crus2_R | Right cerebellar crus II | 2.89 |
| 28 | Cingulate_Ant_R | Right anterior cingulate and paracingulate gyrus | 2.86 |
| 54 | Hippocampus_R | Right hippocampus | 2.87 |
| 32 | Cingulate_Post_R | Right posterior cingulate gyrus | 2.81 |
| 8 | Caudate_R | Right caudate nucleus | 2.77 |
| 24 | Cerebelum_Crus1_R | Right cerebellar crus I | 2.76 |

Supplementary Material 5. Results from the exploratory analysis featuring all regions in the AAL2 atlas. Table showing the brain regions where significant differences (p_FDR_ <0.05) were found between patients and controls in neural activity to the *green* contrast.

**S6. Electrodermal activity**
The supplementary analysis of electrodermal data included all subjects, i.e. 41 patients and 44 healthy controls. Data was missing for one healthy control in one *green* and one *positive* picture trial, the remaining data from this subject was included in the analysis.
Patients differed significantly from healthy controls in their electrodermal reactivity. Healthy controls had a higher electrodermal reactivity to *red* and *green* color cues (t(83)= 2.18 , *p*= 0.03 and t(83)= 2.43, *p*=0.017, respectively). No significant difference between groups was found for reactivity to *negative* and *positive* pictures (*p*= 0.056 and *p*= 0.71, respectively).

**References**

1. Esteban O, Blair R, Markiewicz CJ, Berleant SL, Moodie C, Ma F, et al. fMRIPrep 23.3.1. 2018.

2. Esteban O, Markiewicz CJ, Blair RW, Moodie CA, Ilkay Isik A, Erramuzpe A, et al. fMRIPrep: a robust preprocessing pipeline for functional MRI. Nature Methods. 2019;16:111-6.

3. Gorgolewski K, Burns CD, Madison C, Clark D, Halchenko YO, Waskom ML, et al. Nipype- a flexible, lightweight and extensible neuroimaging data processing framework in Python. Frontiers in Neuroinformatics. 2011;5.

4. Gorgolewski K, Esteban O, Markiewicz CJ, Ziegler E, Ellis DG, Notter MP, et al. Nipype 1.8.6. 2018.

5. Tustison NJ, Avants BB, Cook PA, Zheng Y, Egan A, Yushkevich PA, et al. N4ITK: Improved N3 Bias Correction. Transactions on medical imaging. 2010;29(6).

6. Avants BB, Epstein C.L., Grossman M, Gee JC. Symmetric diffeomorphic image registration with cross-correlation: Evaluating automated labeling of elderly and neurodegenerative brain. Medical Image Analysis. 2008;12:26-41.

7. Zhang Y, Brady M, Smith S. Segmentation of Brain MR Images Through a Hidden Markov Random Field Model and the Expectation-Maximization Algorithm. Transactions on medical imaging. 2001;20(1).

8. Ciric R, Thompson WH, Lorenz R, Goncalves M, MacNicol EE, Markiewicz CJ, et al. TemplateFlow: FAIR-sharing of multi-scale, multi-species brain models. Nature Methods. 2022;19:1568-71.

9. Jenkinson M, Bannister P, Brady M, Smith S. Improved Optimization for the Robust and Accurate Linear Registration and Motion Correction of Brain Images. NeuroImage. 2002;17:825-41.

10. Jenkinson M, Smith S. Global Optimisation Method for Robust Affine Registration of Brain Images. Medical Image Analysis. 2001;5:143-56.

11. Greve DN, Fischl, B. . Accurate and Robust Brain Image Alignment Using Boundary-Based Registration. NeuroImage. 2009;48:63-72.

12. Power JD, Mitra A, Laumann TO, Snyder AZ, Schlaggar BL, Petersen SE. Methods to detect, characterize, and remove motion artifact in resting state fMRI. NeuroImage. 2014;84:320-41.

13. Behzadi Y, Restom K, Liau J, Liu TT. A Component Based Noise Correction Method (CompCor) for BOLD and Perfusion Based fMRI. NeuroImage. 2007;37(1):90-101.

14. Satterthwaite TD, Elliott MA, Gerraty RT, Ruparel K, Loughead J, Calkins ME, et al. An improved framework for confound regression and filtering for control of motion artifact in the preprocessing of resting-state functional connectivity data. NeuroImage. 2013;64(1):240-56.

15. Patriat R, Reynolds RC, Birn RM. An Improved Model of Motion-Related Signal Changes in fMRI. NeuroImage. 2017;144:74-82.

16. Abraham A, Pedregosa F, Eickenberg M, Gervais P, Mueller A, Kossaif J, et al. Machine Learning for Neuroimaging with Scikit-Learn. Frontiers in Neuroinformatics. 2014.

17. Benjamini Y, Yekutieli D. The control of the false discovery rate in multiple testing under dependency. The Annals of Statistics. 2001;29(4):1165-88.

18. Tzourio-Mayozer N, Landeau B, Papathanassiou D, Crivello F, Etard O, Delcroix N, et al. Automated Anatomical Labeling of Activations in SPM Using a Macroscopic Anatomical Parcellation of the MNI MRI Single-Subject Brain. NeuroImage. 2002;15.
